# Click display: a rapid and efficient *in vitro* protein display method for directed evolution

**DOI:** 10.1093/nar/gkad643

**Published:** 2023-08-07

**Authors:** Yu Zeng, Michael Woolley, Karuppiah Chockalingam, Benjamin Thomas, Srishtee Arora, Magnus Hook, Zhilei Chen

**Affiliations:** Department of Microbial Pathogenesis and Immunology, Texas A&M University Health Science Center, Bryan, TX 77807, USA; Department of Microbial Pathogenesis and Immunology, Texas A&M University Health Science Center, Bryan, TX 77807, USA; Department of Microbial Pathogenesis and Immunology, Texas A&M University Health Science Center, Bryan, TX 77807, USA; Interdisciplinary Graduate Program in Genetics and Genomics, Texas A&M University, Houston, TX 77030, USA; Center for Infectious and Inflammatory Diseases, Institute of Biosciences and Technology, Texas A&M University Health Science Center, Houston, TX 77030, USA; Center for Infectious and Inflammatory Diseases, Institute of Biosciences and Technology, Texas A&M University Health Science Center, Houston, TX 77030, USA; Department of Microbial Pathogenesis and Immunology, Texas A&M University Health Science Center, Bryan, TX 77807, USA; Interdisciplinary Graduate Program in Genetics and Genomics, Texas A&M University, Houston, TX 77030, USA

## Abstract

We describe a novel method for *in vitro* protein display—click display—that does not depend on maintaining RNA integrity during biopanning and yields covalently linked protein–cDNA complexes from double-stranded input DNA within 2 h. The display is achieved in a one-pot format encompassing transcription, translation and reverse transcription reactions in series. Stable linkage between proteins and the encoding cDNA is mediated by a modified DNA linker—ML—generated via a click chemistry reaction between a puromycin-containing oligo and a cDNA synthesis primer. Biopanning of a click-displayed mock library coupled with next-generation sequencing analysis revealed >600-fold enrichment of target binders within a single round of panning. A synthetic library of Designed Ankyrin Repeat Proteins (DARPins) with ∼10^12^ individual members was generated using click display in a 25-μl reaction and six rounds of library panning against a model protein yielded a panel of nanomolar binders. This study establishes click display as a powerful tool for protein binder discovery/engineering and provides a convenient platform for *in vitro* biopanning selection even in RNase-rich environments such as on whole cells.

## Graphical Abstract

**Graphical Abstract:**
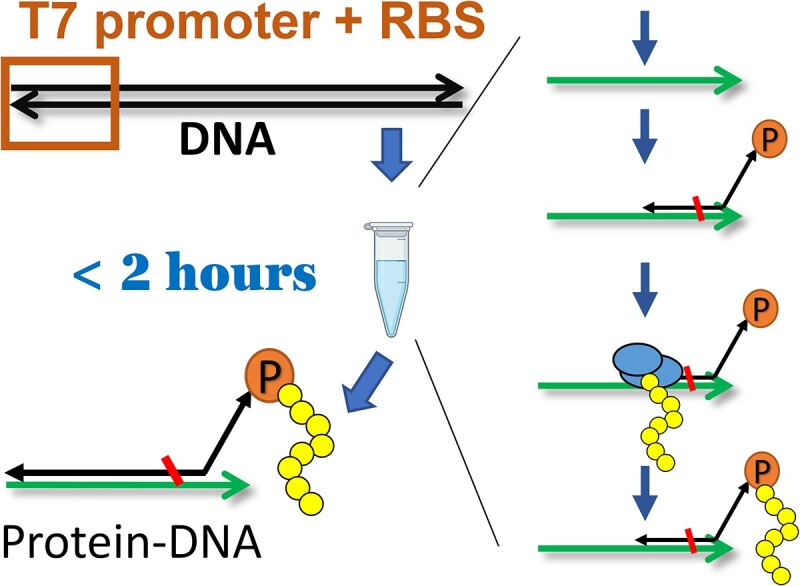

## INTRODUCTION

Rational design of proteins with desirable properties is challenging due to the complexity of a protein's structure-function relationship. Directed evolution, which mimics natural evolution and entails the screening of large libraries of variants to identify those with desired functions, offers a powerful alternative to rational protein design as it does not require *a priori* knowledge of a protein's structure-function relationship. A prerequisite for directed evolution is a link between the protein and its coding DNA/RNA and the choice of display method dictates the size/diversity of the protein library accessible for discovery. Over the years, many cell-based (e.g. yeast/bacterial/phage-display) and cell-free (*in vitro*) protein display methods have been developed. Since cell-free protein display methods are typically able to access a library size (10^12^–10^14^) orders of magnitude larger than cell-based methods (10^6^–10^9^), *in vitro* protein display methods are often preferred for drug development.

One of the earliest cell-free display technologies, mRNA display, takes advantage of the ability of puromycin to enter the ribosomal A site and attach to the nascent peptide. Specifically, a single-stranded DNA (∼30 nt) harboring a puromycin molecule at its 3′ end is ligated to the coding mRNA to enable the formation of protein-mRNA conjugates upon translation(1,2). This method, for the first time, enabled cell-free protein engineering and greatly expanded the library size accessible to directed evolution(1,3). However, major drawbacks of this system are the poor ligation efficiency, making the method highly labor-intensive, and dependence of RNase-free conditions to preserve RNA integrity.

Ribosome display, another *in vitro* protein display technology, utilizes a spacer sequence and a stem loop structure at the 3′ end of the mRNA to ‘trap’ the ribosome, thus bypassing the need for mRNA–DNA ligation (4). Upon translation, this spacer sequence remains attached to the peptidyl tRNA and occupies the ribosomal tunnel, allowing the protein of interest to protrude out of the ribosome. Although ribosome display is procedurally simpler than mRNA-display (5–11), its dependence on mRNA and ribosomes for display confers significant limitations (12): (i) since the ribosome is a large protein–RNA complex, unpredictable interactions between the ribosome and the displayed protein can bias the selection of binder proteins(13); (ii) the peptidyl-tRNA/mRNA/ribosome complex is intrinsically unstable, requiring the selection steps to be performed before reverse transcription (RT), under a high magnesium concentration and at low temperatures (e.g. 4°C)(4,7,14), potentially fostering the selection of variants with restricted biological relevance; (iii) a strictly RNase-free environment is necessary to preserve the integrity of the mRNA during panning, making the success of ribosome display experiments highly sensitive to even small differences in experimental practices and reagents/consumables.

TRAP technology (TRanscription-translation coupled with Association of Puromycin linker) is another *in vitro* display approach developed by Murakami *et al.* that couples mRNA transcription, protein translation and reverse transcription to dramatically reduce the time needed for protein display (12). In TRAP display, a single-stranded DNA linker functionalized with a puromycin molecule at its 3′ end is hybridized to the mRNA in the transcription/translation reaction mixture that lacks Release Factor 1 (RF1). The mRNA contains a UAG STOP codon immediately preceding the mRNA/DNA hybridization region which stalls the ribosome and enables the puromycin to be incorporated into the nascent protein. The display efficiency was later increased by using 2′-Ome-modified DNA linker to suppress promoter-independent transcription and unwanted DNA/RNA duplexes(15). Most recently, Murakami and co-workers created a branched DNA to covalently link the nascent protein with the reverse transcribed first strand cDNA to obviate the need for RNase-free conditions during biopanning (16,17). Although conceptually elegant and simple, it is a non-trivial task to synthesize the branched DNA linker which is not commercially available at the time of this writing and suffers from very low synthesis yield (<0.5–4%) (18).

**Figure 1.**
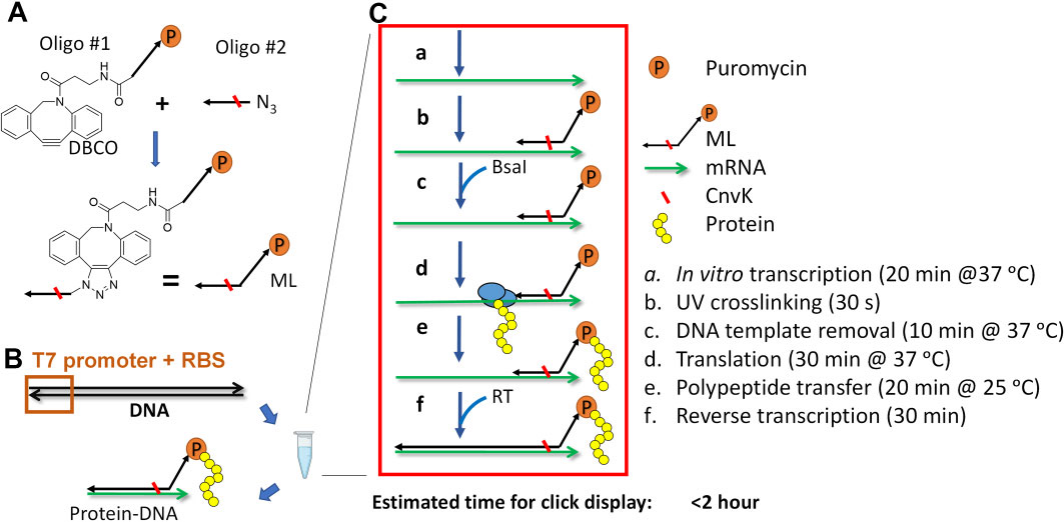
Click display of proteins on their encoding cDNA molecules. (**A**) Design of the modified linker ML. (**B**, **C**) Overview of click display steps. Purified DNA template (1 μg/25 μl reaction) is added directly to the *in vitro* protein synthesis reaction together with ML (final 2 μM). *In vitro* transcription, ML-mRNA crosslinking, *in vitro* translation, and reverse transcription reactions are carried out sequentially in a single tube to yield click-displayed protein–cDNA complexes. Black and green arrows represent single stranded DNA and mRNA, respectively. All arrows point to the 3′ end.

In this study, we describe a simple method for *in vitro* protein display—termed click display—that employs only reagents that are readily available from commercial sources and produces covalently linked protein–cDNA product from coding DNA in a one-pot reaction within 2 h. Covalent linkage of the protein variants and their encoding cDNA molecules is achieved using a modified DNA linker—ML—generated by copper-free click reaction between two oligos (Figure 1A): oligo #1 contains a puromycin molecule at the 3′ end and a click chemistry moiety (i.e. dibenzocyclooctyne (DBCO)) at the 5′ end; oligo #2 is complementary to the 3′ end of the mRNA and harbors a matching click chemistry moiety (i.e. azide (N_3_)) at its 5′ end. The highly efficient and specific click chemistry reaction between DBCO and N_3_ enables the modified oligo ML to be produced with very high efficiency under physiological conditions (19), obviating the need for purification. Oligo #2 also contains a 3-cyanovinylcarbzaole nucleotide (CnvK, red line in Figure 1A), enabling ML to be covalently crosslinked to the mRNA with nearly 100% efficiency upon brief exposure to long wavelength UV (>360 nm) (20,21). Both oligos #1 and #2 are commercially available and the crosslinked mRNA-ML can be efficiently produced using a low-cost UV lamp, allowing click display to be readily performed in any molecular biology lab.

Although in principle any *in vitro* protein synthesis mixture can be used for click display, we have optimized the reaction based on the PURExpress *In Vitro* Protein Synthesis Kit (NEB)(22) which contains all the components needed for *in vitro* transcription and translation. Template DNA harboring a T7 promoter and ribosome binding site (RBS) is added directly to the *in vitro* protein synthesis reaction mixture (1 μg DNA per 25 μl reaction) alongside ML (final 2 μM, Figure 1B). Transcription, ML-mRNA crosslinking, translation, and reverse transcription are carried out sequentially in a one-pot reaction to yield nascent protein–cDNA complexes that can be used directly for biopanning (Figure 1C). Synthesis of protein–cDNA complex from the input DNA template is complete within 2 h (Table 1).

**Table 1.** Overview of one-pot click display of proteins to their encoding cDNA

Components	Vol needed	Final conc./note
Solution A	10 μl	
Solution B	7.5 μl	
RNase inhibitor murine	0.5 μl	
ML (25 μM)	2 μl	2 μM
Template DNA	X μl	80 nM
ddH2O	Y μl	
**Total**	**25 μl**	
• Incubate at 37 °C for 20 min for transcription		
• UV irradiation (30 s)		
BsaI-HF v2 (20 U/μl)	2 μl	
• Incubate at 37°C for 10 min without shaking.		
• Incubate at 37°C for 30 min with shaking for translation.		
• Incubate at room temperature for 20 min without shaking to promote protein transfer.		
EDTA (300 mM)	3 μl	30 mM
• Incubate at room temperature for 5 mins to inactivate the ribosome.		
MgCl_2_ (300 mM)	3 μl	
ddH_2_O	62.5μl	
5× First strand buffer	30 μl	
dNTP (10 mM)	7.5 μl	0.5 mM
DTT (0.1 M)	15 μl	10 mM
SuperScript RT	2 μl	
**Total**	**150 μl**	Expand vol 3–5-fold
• Incubate at 42 or 50°C for 30 min		

The ability of click-displayed libraries to support the selection of rare binders to a desired target protein was demonstrated using two complementary approaches. First, a click-displayed mock designed ankyrin repeat (DARPin) library containing 0.01% of a known enhanced green fluorescent protein (eGFP)-binding DARPin—D_3G86_—(23) was panned against biotinylated eGFP. This approach revealed that 625-fold enrichment of the eGFP-binding DARPin was achieved in a single-round of selection, representing a 6–12-fold enhancement in enrichment efficiency over conventional mRNA-display (24). Next, we displayed a naïve DARPin library with ∼10^12^ variants in a 25-μl click-display reaction and used it to identify a panel of binders to a model target protein—clumping factor A (ClfA)(25)—following six rounds of panning. Several of the selected ClfA binders showed nanomolar target binding affinity as determined by ELISA (Figure 7A).

## MATERIALS AND METHODS

### DNA/mRNA synthesis and purification

The template DNA sequence used for the click display of D_3G86_ is shown in Supplementary Figure S3. For optimizing the reaction condition using mRNA-ML, this DNA template was PCR-amplified using primers 1938 and 2643 (Supplementary Table S1) and use as the template for mRNA synthesis (1 μg per 20 μl reaction, HiScribe™ T7 High-Yield RNA Synthesis Kit (New England Biolabs, E2040S)). At the end of the reaction, 70 μl nuclease-free water, 10 μl 10× DNase I Buffer, and 2 μl DNase I (2 U/μl, New England Biolabs, M0303S) were added and the mixture was incubated at 37°C for another 15 min to degrade the template DNA. The synthesized mRNA was purified using EZNA MicroElute RNA Cleanup Kit (VWR, 101319–190), eluted in nuclease-free water and stored at –80°C in aliquots until use.

To construct a naïve DARPin library for click display, synthetic oligos AR-F2 and AR-R2 (Supplementary Table S1, final 1.6 μM), encoding all the variable regions, were incubated in 1× Isothermal Amplification Buffer (New England Biolabs, B0537S) in the presence of MgSO_4_ (final 6 mM) and dNTP (final 1.4 mM each) at 94°C for 2 min followed by slow cooling (–0.1°C/s) to 65°C before the addition of Bst2.0 (final 320 U/ml). The mixture was incubated at 65°C for ∼30 min to generate double-stranded DARPin variable region (DVR). The synthesized DNA product was purified from agarose gel. The N-cap and C-cap region of D_3G86_ were amplified by PCR using primers 2617/2701 and 2702/2581, respectively, digested with BsaI and ligated to BsaI-digested DVR. The final ligation product (700 bp) was loaded on an agarose gel, and, after gel purification, 650 ng DNA product was recovered. This amount of DNA corresponds to 1.4 pmol (650 ng/(700 bp × 660 g/mol/bp)) or 8.4 × 10^11^ (1.4 pmol × 6 × 10^23^ molecules/mol) different DNA molecules. The entire amount of DNA was used as template for PCR amplification using primers 1938/2643 and Taq polymerase in a total of 3.2 ml of PCR reaction to preserve the library diversity. The final DNA product was purified again on agarose gel and used for click display (1 μg per 25 μl reaction).

### Synthesis of ML and mRNA-ML

Oligo #1 and #2 (Supplementary Table S1) were custom-synthesized by IDT (Integrated DNA Technologies, Inc., IA) and resuspended in DNA duplex buffer (Integrated DNA Technologies, Inc., 11-01-03–01) to achieve a final concentration of 0.5 mM. An equal volume of oligo #1 and oligo #2 were mixed in a thin-wall PCR tube (Fisher Scientific, 14230225) and incubated at room temperature in the dark. The reaction product—ML—was diluted 10-fold in DNA duplex buffer and stored at –80 °C until use. To estimate the reaction efficiency, an aliquot of the reaction mix was removed at 10 min, 1 day or 2 days, diluted 10-fold immediately in water (final 25 μM of each oligo) and stored at –20°C until analysis on a 15% Urea-PAGE gel (Novex, EC6885BOX) and visualized under UV exposure both before and after staining with SYBR Green dye.

mRNA-ML was prepared by incubating purified mRNA (final 1–8 μM) with ML (1:1.2 molar ratio) in DNA duplex buffer in the presence of RNasin Plus RNase inhibitor (final 1 U/μl, Promega, PRN2611) in a thin-wall PCR tube. To maximize the hybridization efficiency between mRNA and ML, the mixture was heated at 94°C for 1 min, slowly cooled to 70°C at –0.1°C/s, incubated at 70°C for 5 min and finally slowly cooled to 25°C at the same rate and held at 25°C for an additional 15 min. After hybridization, the mixture was irradiated under a nail spa UV lamp (SUNUV Gel Nail Light, Model SUNONEWT, Amazon) for 30–90 s. The samples were resuspended in 2xRNA buffer (TBE-Urea Sample Buffer 2×, G-Biosciences, 786-474) and incubated at 70°C for 10 min to denature the mRNA. The crosslinking efficiency was analyzed using 15% and 6% urea–PAGE gels (2 pmol mRNA per lane, Novex™ TBE–urea gels, EC6885BOX, EC6865BOX) in warm 1× TBE buffer (55°C) under a constant voltage of 180 and 100 V, respectively.

### Click display translation optimization

To compare the amount of click displayed product (Figure 3A), different amount of input mRNA-ML was added to the translation reaction mixture (New England Biolabs, PURExpress *in vitro* protein synthesis, E6800). The mixture was incubated at 37°C for 30 min with shaking followed by 20 min incubation at room temperature without shaking. After translation, RNase A (final 1 μg/μl, Omega Bio-tek, AC118) was added to degrade the mRNA, and the mixture was incubated at 37°C for another 30 min. For gel analysis, the samples were first diluted 2-fold in 2× gel shift buffer (50%(v/v) formamide, 125 mM Tris–HCl pH 6.8, 10 mM DTT, 20 mM EDTA and 2% (w/v) SDS)(15), heat inactivated at 95°C for 5 min, and then analyzed on 12% SDS-PAGE gels (Bio-Rad, Cat# 4561043). After electrophoresis, the gel was visualized under UV exposure without staining.

To evaluate the click display efficiency from DNA template (Figure 3B), 1 μg (600 nM) template DNA encoding D_3G86_, T7 promoter and terminator (Supplementary Figure S3) was added directly to 1 reaction volume of PURExpress *in vitro* protein synthesis reaction together with 2 or 3 μM of ML. The reaction mixture was incubated at 37°C for 20 min to allow for mRNA transcription before the addition of BsaI-HF (final 1.6 U/μl, New England Biolabs, R3535) to cleave the template DNA and terminate the transcription. The mixture was then incubated at 37°C for 10 min without shaking, for DNA digestion, and 30 min with vigorous shaking for protein translation, and was finally incubated for another 20–30 min at room temperature to allow for protein transfer. RNase A (Omega Bio-Tek, AC118, final 1μg/μl) was then added to degrade the mRNA and the mixture was diluted 2-fold in 2× gel shift buffer before being analyzed on 12% SDS-PAGE gels (Bio-Rad, 4561043). After electrophoresis, the gels were visualized under UV directly without staining.

### Click display reverse transcription condition optimization

Click display was carried out as described above using mRNA-ML (final 0.5 μM, 25 μl reaction) as the template. After translation, EDTA (final 30 mM, ThermoFisher Scientific, AM9260G) was added to the reaction to inactivate the ribosome (15), followed by MgCl_2_ (300 mM × 3 μl) to neutralize the excess EDTA. The reaction volume was then expanded 3–5-fold before the addition of dNTP (final 0.5mM), SuperScript II (SS-II, final 2.7 U/μl, Invitrogen) or SuperScript IV (SS-IV, final 2.7 U/μl, Invitrogen) reverse transcriptase and the appropriate reaction buffer. The mixture was incubated at 42°C (for SS-II) or 50°C (for SS-IV) for 30 min, diluted 100-fold in ddH2O and the amount of the synthesized cDNA was quantified using Forget-Me-Not™ EvaGreen® qPCR Master Mix (Biotium, 31045) with primers 1892 and 2488 (Supplementary Table S1) in a qPCR instrument (Bio-Rad, C1000 Touch™ Thermal Cycler and CFX 96 Real-Time PCR Detection Systems). After the reaction, the PCR product was also analyzed on 1% agarose gels to ensure the amplification of the correct target DNA.

### Protein expression and purification

ClfA, eGFP (amino acid sequences in Supplementary Figure S3) and all the DARPin molecules were expressed in *Escherichia coli* Bl21 (DE3) cells in LB medium and purified using gravity Ni-NTA agarose beads following the standard procedure. Protein purity was determined using 12% SDS-PAGE gels.

### Streptavidin pull-down assay

eGFP and ClfA were biotinylated using EZ-Link Sulfo-NHS-LC-Biotin (1:4 molar ratio, Fisher Scientific, PI21335). Click-displayed D_3G86_ was diluted 10-fold in blocking buffer SBTD0.3 (StartingBlock™ Blocking Buffer (Thermo Fisher Scientific, 37578) supplemented with 0.05% Tween-20, and 0.3 mg/ml ssDNA (deoxyribonucleic acid sodium salt from salmon testes, Sigma, D1626)) in the absence or presence of 200 nM biotinylated eGFP. The mixture was incubated at room temperature for 1 h before the addition of streptavidin-coated magnetic beads (Dynabeads™ MyOne™ Streptavidin T1, Thermo Fisher Scientific, 65-601) to pull-down the biotinylated eGFP and the associated cDNA. The beads were washed 8 times with SBTD0.3. After the final wash, the beads were resuspended 1x Taq ThermoPol^®^ Reaction Buffer (New England Bio Labs, B9004S) and the amount of cDNA associated with the beads was quantified using Forget-Me-Not qPCR kit with primers 1892 and 2488.

To purify click-displayed product via IMAC, the reaction mixture after reverse transcription was either buffer-exchanged into PBS (50 mM NaH_2_PO_4_, 300 mM NaCl) (26) via ultrafiltration using 30 kDa MWCO Amicon columns (Amicon™ Ultra-0.5 Centrifugal 30kDa MWCO Filter Units, MilliporeSigma™, UFC503024) or diluted 10-fold in PBS + 10 mM imidazole to reduce the DTT concentration before the addition of Ni-NTA agarose bead slurry (company Pierce™ Ni-NTA Magnetic Agarose Beads, 78605). His-tagged proteins were purified following the standard protocol.

### Library enrichment

To estimate the enrichment efficiency, 1 μg of DNA encoding a naïve N3C DARPin library was mixed with 0.1 ng of DNA encoding D_3G86_ and used as template for click display (Table 1). After reverse transcription, the mixture was purified by one-step IMAC and incubated with 200 nM biotinylated eGFP at room temperature for 1 hour. Streptavidin T1 or neutravidin-coated magnetic beads (Cytiva SpeedBeads Magnetic Neutravidin Coated Particles, Fisher Scientific, 09 981 154) were added to the mixture to pull down biotinylated eGFP and the associated click-displayed product. The beads were washed 3–8 times for rounds 1 to 3. After the final wash, the beads were resuspended in 1x Standard Taq buffer and the cDNA was amplified by PCR using Taq polymerase (NEB, M0267S) and primers 1938 and 2643, which contain the T7 promoter and terminator region, respectively. The PCR product was used as the template for the subsequent round of panning after gel purification.

To enrich binders to ClfA, click-displayed naïve DARPin library made from 1 μg of DNA was used. This library was purified by IMAC and incubated with biotinylated ClfA (final 200 nM). ClfA-binders were pulled down using streptavidin- or neutravidin-coated beads in alternative rounds.

### Next-generation sequencing (NGS)

NGS samples were prepared by amplifying the PCR product from different rounds of panning (R0-R3) using NGS primers containing the Illumina adapter sequences **(**Supplementary Table S1). The different rounds were distinguished using a two-nucleotide barcode inserted between the Illumina adapter and the complementary region of the primers. The selected cDNA from each round was amplified with its respective primer pair using Q5 High-Fidelity Polymerase (New England Biolabs, M0491). Each round was then purified using the ZymoClean Gel DNA Recovery Kit (Zymo Research), after which 150 ng of the DNA from each round was combined. Samples were sequenced using a Mi-Seq Next-Generation Sequencing system from Amplicon-EZ (Azenta Life Sciences). Paired-end reads were merged using FLASH(27). Sequences were converted to FASTA format, translated, and analyzed using SeqKit (28).

### ELISA

The ELISA experiment was carried out essentially as described previously(29). Briefly, the MaxiSorp immune plates (Nunc MaxiSorp plates, Fisher Scientific, 50-712-278) was coated with ClfA (4 μg/ml) over night. All DARPins contain Myc- and His-tag at the N-terminus (Supplementary Figure S3). The next day, after blocking the plate with DPBSTB (DPBS with 0.1% Tween-20 and 2% BSA), serially diluted IMAC-purified DARPin was added to the plate. After incubation and washing, well-bound DARPin proteins were detected using mouse anti-c-Myc antibody (Invitrogen, 13–2500) and horseradish peroxidase (HRP)-conjugated goat anti-mouse antibody (Jackson Immuno Research, 115-035-146) followed by color development using TMB (3,3′, 5,5″-tetramethylbenzidine) substrate.

### Model generation

The 3D model of D_1F8_ was generated using AlphaFold 2.2.0 (30,31) at Texas A&M High Performance Research Computing (HPRC) facility. The five lowest ranked models were nearly identical, and the lowest ranked model was used in this study.

## RESULTS

### Synthesis of ML and mRNA-ML

**Figure 2.**
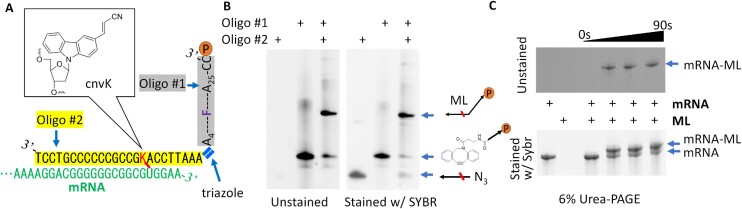
ML synthesis by copper-free click chemistry. (**A**) Design of oligo #1 and oligo #2. (**B**) ML can be efficiently synthesized under ambient conditions. Oligo #1 and oligo #2 were mixed in at an equimolar ratio (250 μM of each oligo) and incubated at room temperature for 10 min. 100 pmol of each oligo, either alone or following the 10-min click reaction, was analyzed on a 15% urea–PAGE gel that was first visualized under UV, and then stained with SYBR Green and visualized again. Only oligo #1 and ML are visible under UV without dye staining. (**C**) ML can be efficiently crosslinked to mRNA. After hybridization, the mixtures of mRNA and ML were irradiated under UV for 30, 60 or 90s before being analyzed on a 6% urea–PAGE gel. Only ML-crosslinked mRNA is visible on unstained gels. Due to its small size, ML is not visible on the 6% Urea-PAGE gel but can be seen on a 15% urea–PAGE gel (Supplementary Figure S2).

The puromycin-containing linker ML was produced by reacting oligo #1 and oligo #2 (Figure 2A). Oligo #1 contains a fluorescently labeled nucleotide (TAMRA) to facilitate the subsequent gel analysis. Oligo #2 is complementary to the mRNA and can be crosslinked to the mRNA via the cnvK (K) nucleotide upon UV irradiation. These oligos were mixed at an equimolar ratio (250 μM each oligo) in DNA duplex buffer (IDT) and incubated at room temperature. After only 10 min of incubation, nearly all the input oligos have reacted to yield ML (Figure 2B, Supplementary Figure S1). The reaction efficiency is estimated to be >90% by densitometry analysis. To simplify the calculation of the ML input amounts, for subsequent steps, we assumed 100% ML synthesis efficiency.

Next, we confirmed the ability of ML to be specifically crosslinked to mRNA. Purified mRNA (final 1 μM), whose 3′ end is complementary to oligo #2 (Figure 2A), was mixed with ML at 1:1.2 molar ratio in DNA duplex buffer in a thin-walled PCR tube (20 μl total). The mixture was heated to 94°C for 1 min to denature the mRNA and then slowly cooled to 25°C for hybridization. Finally, the mixture was irradiated under a nail spa UV lamp for 30, 60 and, 90 s and analyzed on Urea-PAGE gels. Over 70% of the input mRNA was crosslinked to ML after 90 seconds UV irradiation as determined by densitometric analysis, with free ML being nearly completely depleted (Figure 2C, Supplementary Figure S2). Although the crosslinking efficiency was marginally higher with 90 s UV irradiation, to maximally preserve the mRNA integrity, 30 seconds irradiation time was selected for all subsequent experiments. To simplify the calculation of mRNA-ML input amounts for subsequent steps, we assumed that all the input mRNA is converted to mRNA-ML.

### Optimization of conditions for protein display

**Figure 3.**
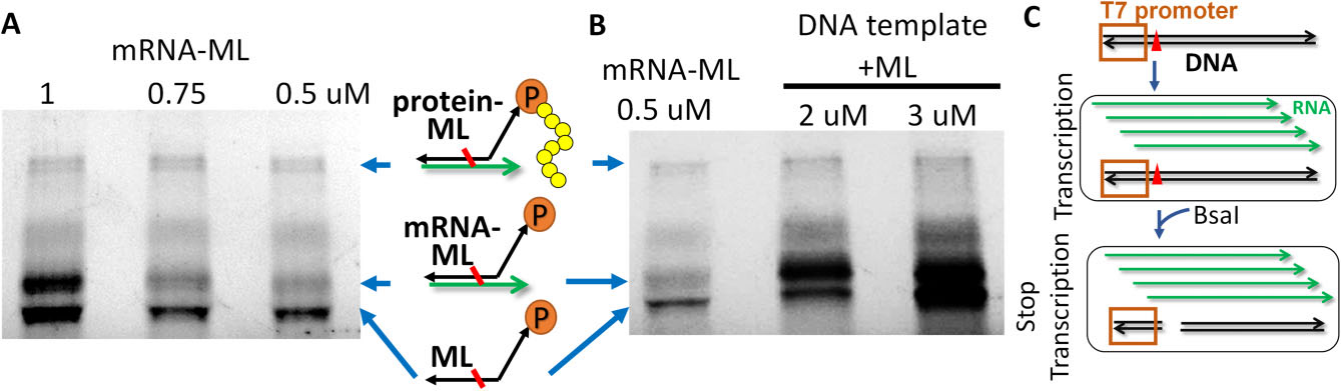
Protein display. (**A**) Comparison of protein display efficiency of mRNA-ML at different concentrations. The product was analyzed on 12% SDS-PAGE gels and visualized under UV without staining. Only ML (containing the fluorescent oligo #1) and protein covalently linked to ML are visible on the gel. p.mRNA-ML: partially digested mRNA-ML. (**B**) Comparison of the protein display efficiency of reactions containing 1 μg of DNA template and 2 or 3 μM of ML to that with 0.5 μM of mRNA-ML. (**C**) Schematic for terminating the transcription reaction. Red triangle represents the BsaI recognition site. Black and green arrows indicate single stranded DNA and mRNA, respectively.

To demonstrate the ability for protein display, mRNA-ML was used as template for protein translation using the PURExpress *In Vitro* Protein Synthesis Kit (NEB). Considering that each PURExpress *in vitro* protein synthesis reaction contains 2.4 μM of ribosome (60 pmol per 25 μl reaction), and that ribosomes in association with the mRNA are not actively released due to mRNA-ML crosslinking at the cnvK site, we compared the yield of protein display with 0.5, 0.75 and 1 μM mRNA-ML. The protein translation reaction was carried out at 37°C for 30 min followed by another 20 min incubation at room temperature to facilitate the transfer of the nascent protein to puromycin(32). Due to the large size of mRNA, it is difficult to distinguish free mRNA and mRNA crosslinked to ML and protein. To simplify the gel analysis, RNase A was added to the reaction mixture to degrade the single-stranded mRNA. Similar amounts of protein-ML were observed in all reactions, suggesting that the display efficiency is likely saturated at 0.5 μM mRNA-ML (Figure 3A). Since mRNA in duplex with ML is partially protected from RNase A digestion, both ML and partially digested mRNA-ML (p.mRNA-ML) are visible on the gel. In the reaction containing 1 μM mRNA-ML, a large amount of p.mRNA-ML is also visible on the gel, indicating that excess of mRNA-ML is present at this condition. Densitometry analysis showed that the intensity of the band corresponding to protein-ML in the 0.5 μM lane is ∼20% of the total ML input, corresponding to 1.5 × 10^12^ mRNA molecules per 25 μl reaction (0.5 μM × 25 μl × 20% × *N*_A_, *N*_A_ = 6 × 10^23^ molecules/mol). This value represents the upper limit of the library diversity per 25 μl reaction.

In order to avoid handling purified mRNA which is highly sensitive to RNase contamination, we next optimized the reaction conditions to enable DNA to be used directly as the template for click display. DNA template (2 pmol per 25 μl reaction or 80 nM) and ML (final 2 and 3 μM) were added directly to the *in vitro* protein synthesis reaction and the transcription reaction was carried out at 37°C for 20 min before being UV irradiated for 30 s. Excess ML was used in this reaction to compensate for the anticipated reduced hybridization efficiency between ML and nascent mRNA due to the lack of the heat denaturation and slow cooling steps. Since mRNA synthesized after UV irradiation will not be crosslinked to ML but can potentially compete with the crosslinked mRNA-ML for ribosomes in the reaction, the restriction enzyme BsaI, which cleaves the DNA template immediately after the T7 promoter (Supplementary Figure S3), was added to the mixture to terminate the transcription reaction (Figure 3C). The mixture was then incubated at 37°C for 40 min to allow both BsaI digestion and protein translation, and then treated with RNase A before electrophoresis. The amount of click-displayed protein appeared to be similar regardless of the amount of the ML (Figure 3B) and is comparable to the control reaction using 0.5 μM mRNA-ML as the template, indicating that DNA template can be used directly for click display without compromising the protein display efficiency. A large ML band was visible on the gel, especially in the reaction containing 3 μM ML, suggesting that the concentration of ML is likely in excess at this condition.

### Optimization of conditions for reverse transcription

**Figure 4.**
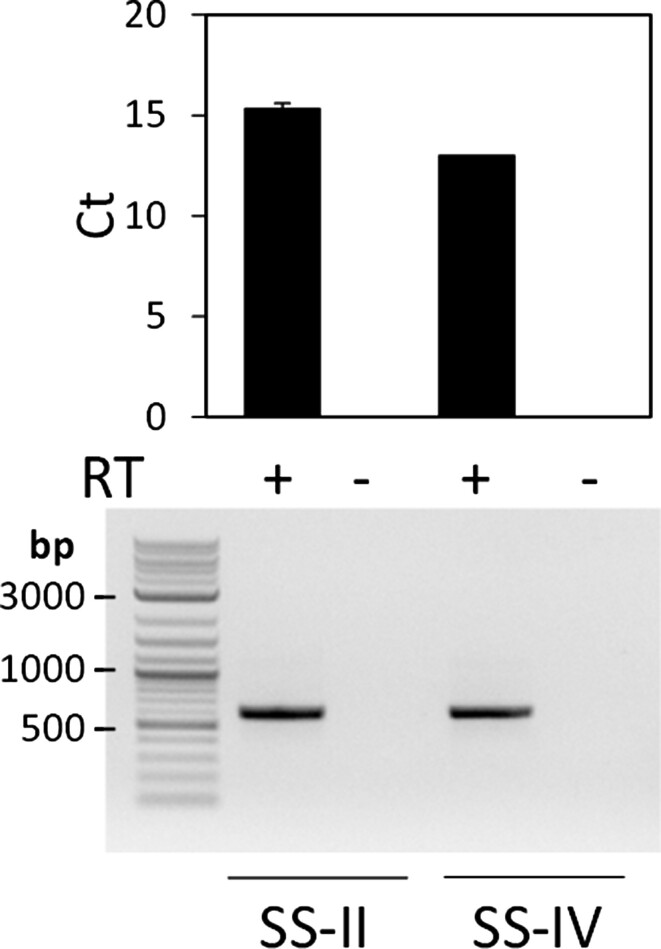
Confirmation of one-pot reverse transcription reaction. RT: Reverse Transcriptase. SS-II: SuperScript II; SS-IV: SuperScript IV. Ct: cycle threshold. The qPCR product was further analyzed on a 1% agarose gel.

We next optimized the final component of the click display reaction series—reverse transcription. To avoid mistaking the input DNA template for the synthesized cDNA, click display was carried out using mRNA-ML (final 0.5 μM) as the template in these studies. At the conclusion of translation, EDTA (final 30 mM) was added to inactivate the ribosomes(15) followed by an equimolar amount of MgCl_2_ to neutralize the EDTA. The mixture was then diluted 3-fold before the reverse transcription reaction. We compared the cDNA synthesis efficiency of SuperScript II (SS-II) and SuperScript IV (SS-IV) whose optimum reaction temperatures are 42°C and 50°C, respectively. The amount of the synthesized cDNA was quantified using qPCR. To ensure that the measured cycle threshold (Ct) values correspond to the amplification of the target DNA, the reaction mixture after qPCR was also analyzed via electrophoresis. The amplified PCR product was present only in samples incubated in the presence of reverse transcriptase (RT) (Figure 4), confirming the synthesis of cDNA under these conditions. Similar amounts of cDNA were present in both reactions, with SS-IV offers a slight advantage in the synthesis efficiency (slightly lower cycle threshold (Ct) value).

### Evaluation of target binding ability of a click-displayed protein

**Figure 5.**
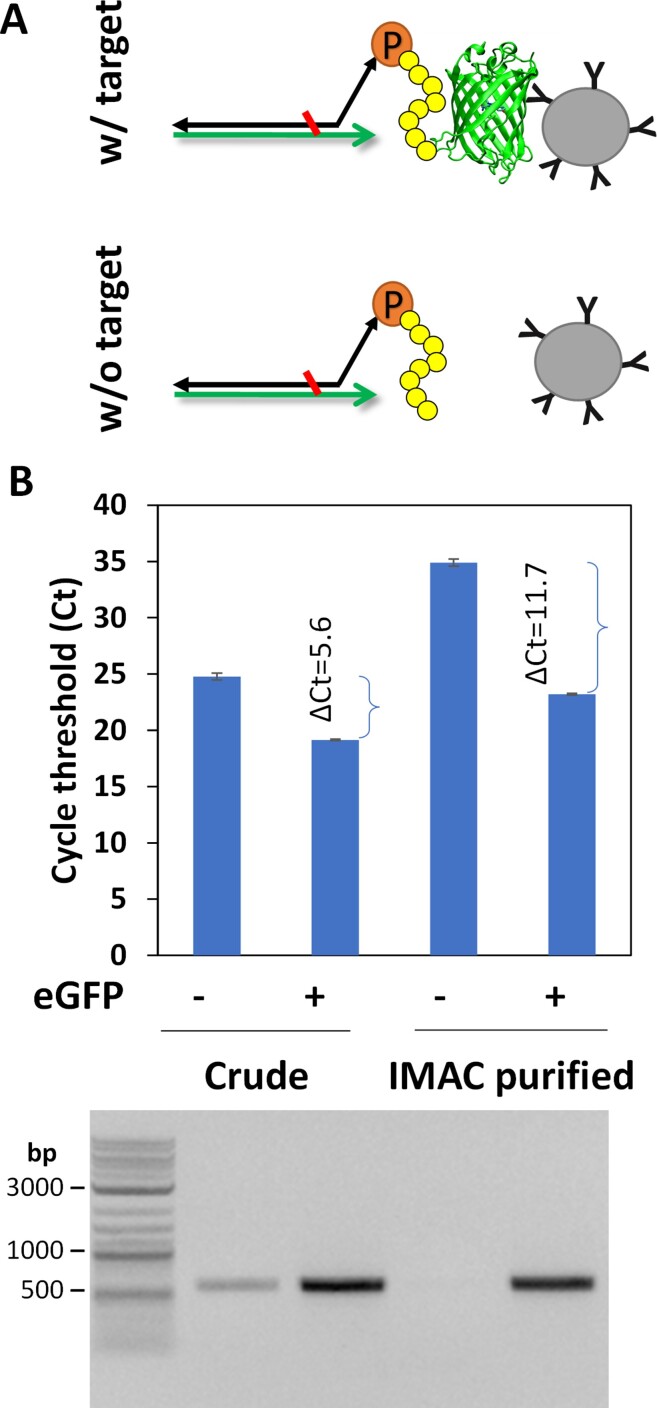
Pull-down of click-displayed anti-GFP DARPin D_3G86_. (**A**) Scheme of the pull-down experiment. Click displayed D_3G86_-cDNA were incubated with streptavidin beads either in the presence or absence of the biotinylated target GFP. (**B**) qPCR quantification of either the crude (unpurified) or IMAC-purified D_3G86_-cDNA complex pulled down by the streptavidin beads. One representative experiment with the mean value of two technical replicates ± standard error is shown. Agarose gel electrophoresis of the PCR products from one of the reactions is shown beneath the corresponding bar graph.

The final one-pot transcription, translation, and reverse transcription reaction protocol is summarized in Table 1. To evaluate the efficiency of click display, we displayed DARPin 3G86 (D_3G86_), which strongly binds eGFP (*K*_D_ ∼ 9 nM)(23), and determined its ability to be pulled down by biotinylated eGFP in a streptavidin pull-down assay (Figure 5A). Briefly, click-displayed D_3G86_ was diluted 10-fold in blocking buffer with or without biotinylated eGFP (final 200 nM). After 30 min incubation at room temperature, streptavidin-coated magnetic beads were added to the mixture to pull-down biotinylated eGFP and the associated click-displayed D_3G86_.

A much larger amount of cDNA was pulled-down in samples incubated in the presence of eGFP than that in buffer alone, as manifested in the significantly lower cycle threshold (Ct) values in quantitative PCR (qPCR, Figure 5B) analysis, confirming that click displayed D_3G86_ is active and retains the ability to associate with eGFP. To ensure that the amplification is specific, the qPCR product was analyzed on agarose gels. A faint band of the amplified cDNA was visible in the sample incubated in the absence of eGFP, likely resulting from non-specific association of the parental template DNA with the beads.

To further reduce the background signal, we exploited the 6xHis tag at the N-terminus of D_3G86_ and purified the click-displayed D_3G86_ product via one-step immobilized metal affinity chromatography (IMAC) prior to incubation with eGFP or buffer. IMAC purification of the input click-displayed protein significantly reduced the background signal resulting in further elevated Ct values for samples incubated in buffer alone and the reduction of PCR product on agarose gels (Figure 5B). Although the Ct values for samples incubated in the presence of eGFP also increased slightly, the ΔCt between samples incubated with and without eGFP increased significantly from 5.6 (original) to 11.7 (after IMAC purification). This result indicates that IMAC purification can efficiently reduce the background signal from click display. The dsDNA template present in the click reaction is likely responsible for the background signal.

### Mock library enrichment

**Figure 6.**
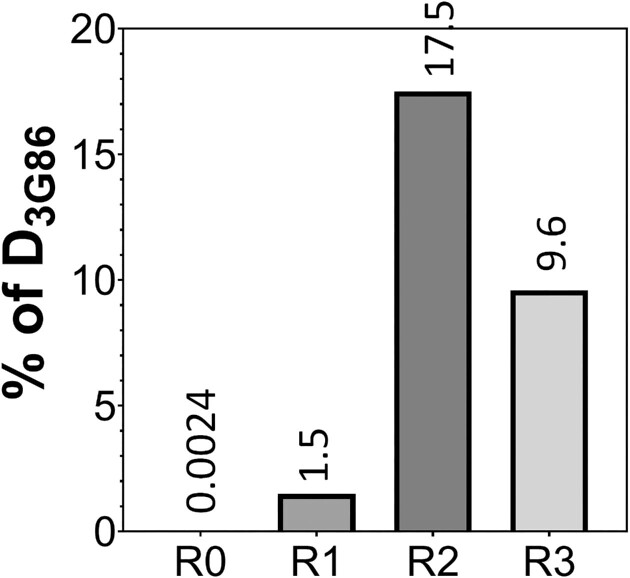
The frequency of D_3G86_ sequence in each round during the mock enrichment.

To evaluate the efficiency of click display to support the selection of rare target protein binders from a large library, we carried out a mock library enrichment experiment. DNA encoding the eGFP-binding DARPin D_3G86_ (0.1 ng) was mixed with a naïve DARPin library (1 μg) at 1:10 000 molar ratio and used as the template for click display. The click displayed product was purified via IMAC and underwent standard panning/selection against biotinylated eGFP (final 200 nM). Streptavidin and neutravidin beads were used to capture the eGFP-binding DARPin(s) in alternative rounds. To quantify the amount of D_3G86_ present in the output from different rounds of selection, approximately 150 ng of the final PCR product from each round was sequenced with a Mi-Seq Next-Generation Sequencing system yielding a dataset with 149768 full-length protein sequences. The frequency of D_3G86_ increased from 0.0024% in R0 to 1.5% in R1 and peaked at 17.5% in R2 (Figure 6). The enrichment efficiency from R0 to R1 is estimated to be 625-fold (1.5%/0.0024%), which is 6–12-fold higher than conventional mRNA-display(24). Somewhat unexpectedly, the frequency of D_3G86_ decreased in R3 to 9.6%. We suspect this phenomenon may be due to the selection of other eGFP-binding DARPins from the library that may out-compete D_3G86_ for eGFP binding. Nevertheless, these results confirm the ability of click display to rapidly enrich binders to a desired target protein.

### Discovery of DARPin binders to a model protein using click display

Next, we used a click-displayed synthetic DARPin library to pan for binders to a model target protein—clumping factor A (ClfA), an *S. aureus* surface virulence protein(25,33). A click-displayed DARPin library prepared from 1 μg of DNA template (∼10^12^ different molecules) was purified via IMAC and underwent standard selection against biotinylated ClfA (final 200 nM). After five rounds of panning, significantly more DARPin–cDNA complexes were pulled down by streptavidin beads incubated in the presence of the target protein ClfA, which is manifested as a higher ΔCt (Supplementary Figure S4A). The PCR product from Round 6 was subcloned into the pET28a expression vector and used to transform BL21(DE3) cells. One hundred and eighty-eight (two 96-well plates minus 4 control wells per plate) clones were picked and grown in LB. The next day, the cell lysates after protein induction were harvested and their ability to bind ClfA was determined via ELISA. Overall, >50% of the clones showed >3 standard deviations above the background binding to ClfA, with >30% showing >10 standard deviations above the background binding (Supplementary Figure S4).

**Figure 7.**
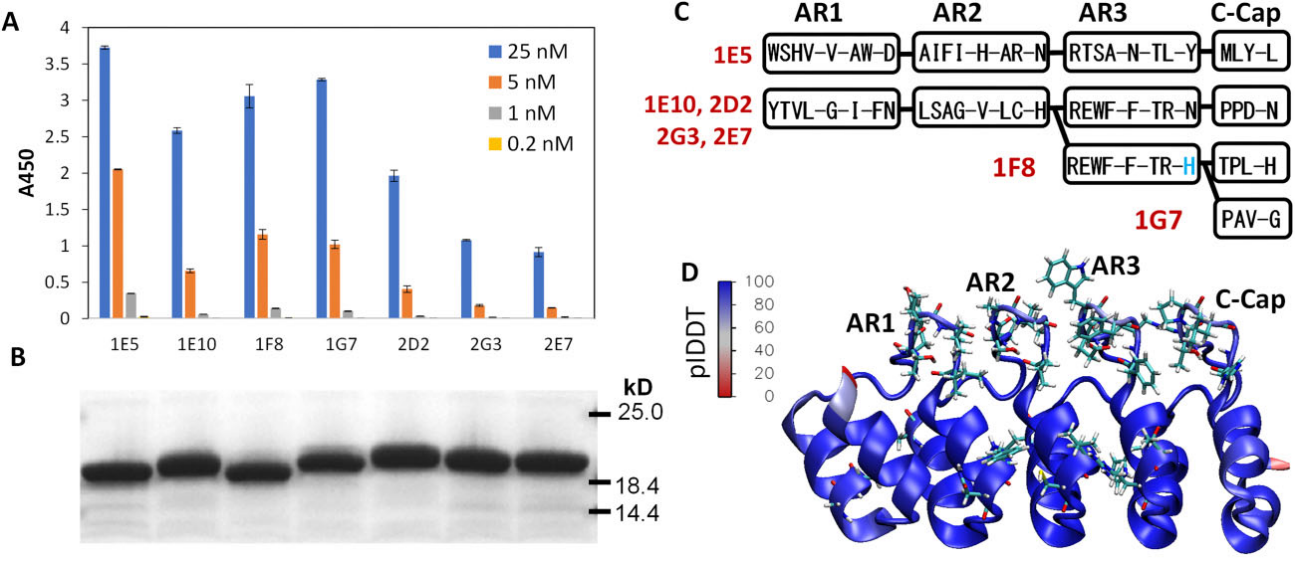
Characterization of ClfA-binding DARPin clones. ClfA-binding affinity measurement by ELISA (**A**) and SDS-PAGE (**B**) of IMAC-purified DARPins selected from six rounds of panning on ClfA. Data points are the mean ± s.d. from one of two representative experiments carried out in duplicate. (**C**) Illustration of the sequences in the variable residues of the different DARPins. (**D**) AlphaFold2-predicted structure of D_1F8_ colored based on each residue's pLDDT value. Variable residues comprising the binding interface (randomized during library creation) are shown in stick form. The pLDDT values (indication of prediction confidence) for residues within the variable loops are much lower than those within the scaffold.

Seven clones that showed strong ClfA-binding when applied as crude cell lysates were purified via IMAC and re-evaluated for binding to ClfA via ELISA (Figure 7A, B). The sequences of these clones were found to be distinct although they cluster into two large families, with DARPin 1E5 (D_1E5_) adopting a different set of target-binding residues than all the other clones. Clones D_1E10_, D_2D2_, D_2G3_ and D_2E7_ share the identical target-binding residues but contain different point mutations in the scaffold (Figure 7C, D, Supplementary Figure S5). The very different ClfA-binding ability of D_1E10_ and D_2E7_ highlights the influence of framework residues on target binding. To evaluate the diversity, the PCR product from R6 was subjected to next-generation sequencing (NGS). We obtained a total of 27026 full-length DARPin sequences, among which D_2G3_ was the most abundant, representing 627 reads (2.3%, Supplementary Figure S6). This result shows that significant polyclonal diversity remains in the click-display-selected pools even after 6 rounds of panning. Since the diversity of the naïve library (estimated to be ∼10^12^) greatly exceeds the capacity of NGS, we did not sequence this initial library. The successful enrichment of ClfA-binding DARPins, as well as the preservation of high library diversity after 6 rounds of enrichment, underscores the power of click display for protein binder discovery.

## DISCUSSION

Current *in vitro* protein display methods are complex, labor-intensive, and often require strictly RNase-free conditions for purification and/or library panning, putting them out-of-reach for many researchers. For example, RNA is used to link the displayed protein to its coding sequence in both mRNA-display and ribosome-display, limiting their applications to highly purified and RNase-free targets (1,4,7,14). Although a recently developed *in vitro* display method, TRAP display, enables simultaneous mRNA transcription and protein translation, it requires a branched oligo that is not commercially available (Integrated DNA Technologies, Inc. does not offer synthesis of the branched oligos at the time of this manuscript preparation) and whose synthesis is non-trivial and inefficient (<0.5% to 4%,)(18), severely limiting the utility of this approach.

In this study, we developed an alternative *in vitro* protein display technology—click display—that enables the production of covalently linked protein–cDNA complex from double-stranded input DNA in a one-pot reaction within 2 h. A modified oligo linker (ML) with two 3′ ends—one functionalized with puromycin and the other a primer for cDNA synthesis—was generated using copper-free click chemistry and was used to link the coding cDNA and the displayed protein. The constituents of ML, Oligo #1 and Oligo #2, are commercially available (*e.g*. IDT) and no purification step is needed for ML synthesis. The estimated cost of ML is ∼$4 per 25 μl *in vitro* protein synthesis reaction. Since the protein is covalently linked to the cDNA, there is no need to maintain RNase-free conditions during biopanning, allowing selections to be potentially carried out in RNAse-rich environments such as on whole cells.

Using a previously engineered eGFP-binding DARPin—D_3G86(23)_—and next-generation sequencing (NGS), the enrichment efficiency of click-displayed libraries was estimated to be 625-fold per round, which is 6–12-fold higher than the conventional mRNA-display techniques (24), demonstrating a high protein display efficiency of click display.

A naïve DARPin library was used to enrich binders to a model protein: ClfA(25). Click displayed DARPin library was prepared from 1 μg of DNA template in 25 μl translation reaction, corresponding to a library size of ∼10^12^. A high-throughput ELISA screen of 188 randomly picked DARPin clones from the Round 6-selected library showed a >50% hit rate for ClfA binders (Supplementary Figure S4B). All seven of the randomly picked clones showed nanomolar ClfA-binding affinity based on ELISA (Figure 6A) and have different sequences, although some pf the sequences differ only by substitutions in DARPin framework region (Supplementary Figure S5). NGS confirmed that the enriched library retains high diversity even after six rounds of panning (Supplementary Figure S6), which indirectly corroborates the large library diversity in the initial rounds.

In conclusion, we report the development of a powerful new tool for *in vitro* protein display and protein engineering—click display—that does not demand an RNAse-free cleanliness standard for panning success and requires only readily available reagents for implementation. The utility of click display was demonstrated through the discovery a panel of novel binders to a *S. aureus* surface virulence protein, clumping factor A, from a synthetic DARPin library with ∼10^12^ distinct members. Although a DARPin library was used in this study, click display can in principle be used to display/engineer any protein amenable to *in vitro* translation. We believe that click display has the potential to accelerate the directed evolution of diverse binders to desired targets.

## Supplementary Material

gkad643_Supplemental_Files

## Data Availability

Next Generation Sequencing data is available in the OSF repository (DOI: https://doi.org/10.17605/OSF.IO/X35FY).
