# Correction to ‘Click display: a rapid and efficient in vitro protein display method for directed evolution’

**DOI:** 10.1093/nar/gkae1172

**Published:** 2024-11-18

This is a correction to: Yu Zeng, Michael Woolley, Karuppiah Chockalingam, Benjamin Thomas, Srishtee Arora, Magnus Hook, Zhilei Chen, Click display: a rapid and efficient in vitro protein display method for directed evolution, Nucleic Acids Research, Volume 51, Issue 16, 8 September 2023, Page e89, https://doi.org/10.1093/nar/gkad643.

The authors wish to correct several errors in their article.

1. The positions of N_3_ and DBCO for Oligo #1 and Oligo #2 in Figure 1A are incorrect. A corrected figure is shown below.

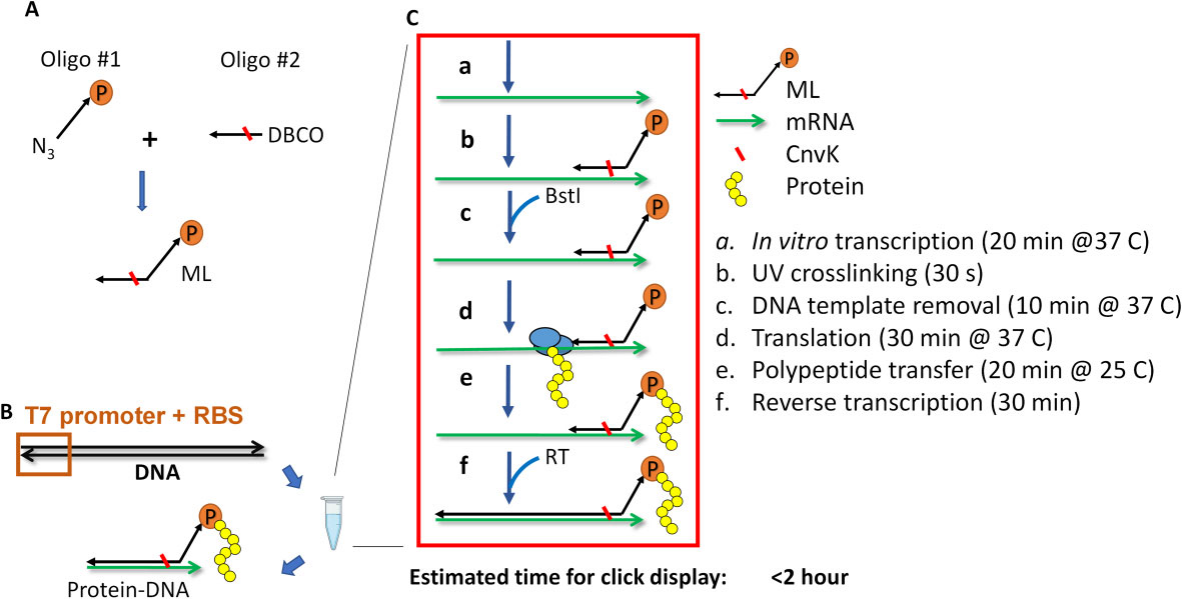

2. The positions of N_3_ and DBCO for Oligo #1 and Oligo #2 in Figure 2B are incorrect. A corrected figure is shown below.

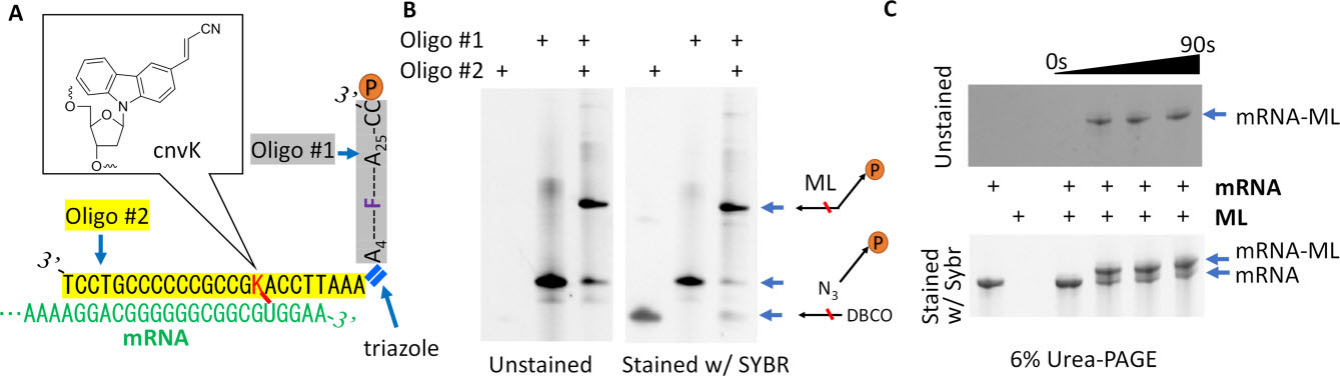

3. The names of Oligo #1 and Oligo #2 in Table S1 are incorrect. The correct names are shown below:

**Table:** 

Name	Sequence	Vendor
Oligo #1	/5AzideN/AAAA/i5-TAMK/AAAAAAAAAAAAAAAAA AAAACC/3Puro/	IDT
Oligo #2	/5DBCON/AAATTCCA/i3Cyan/GCCGCCCCCCGTCCT	IDT
4. The description of Oligo #1 and Oligo #2 in the Introduction is incorrect.
  Original text:
  … oligo #1 contains a puromycin molecule at the 3′ end and a click chemistry moiety (i.e. dibenzocyclooctyne (DBCO)) at the 5′ end; oligo #2 is complementary to the 3′ end of the mRNA and harbors a matching click chemistry moiety (i.e. azide (N3)) at its 5′ end.
  Corrected text:
  … oligo #1 contains a puromycin molecule at the 3’ end and a click chemistry moiety (i.e. **azide (N_3_)**) at **its** 5’ end; oligo #2 is complementary to the 3’ end of the mRNA and harbors a matching click chemistry moiety (i.e. **dibenzocyclooctyne (DBCO)**).
5. The positions of N_3_ and DBCO for Oligo #1 and Oligo #2 in Figure S1 are incorrect. A corrected figure is shown below.

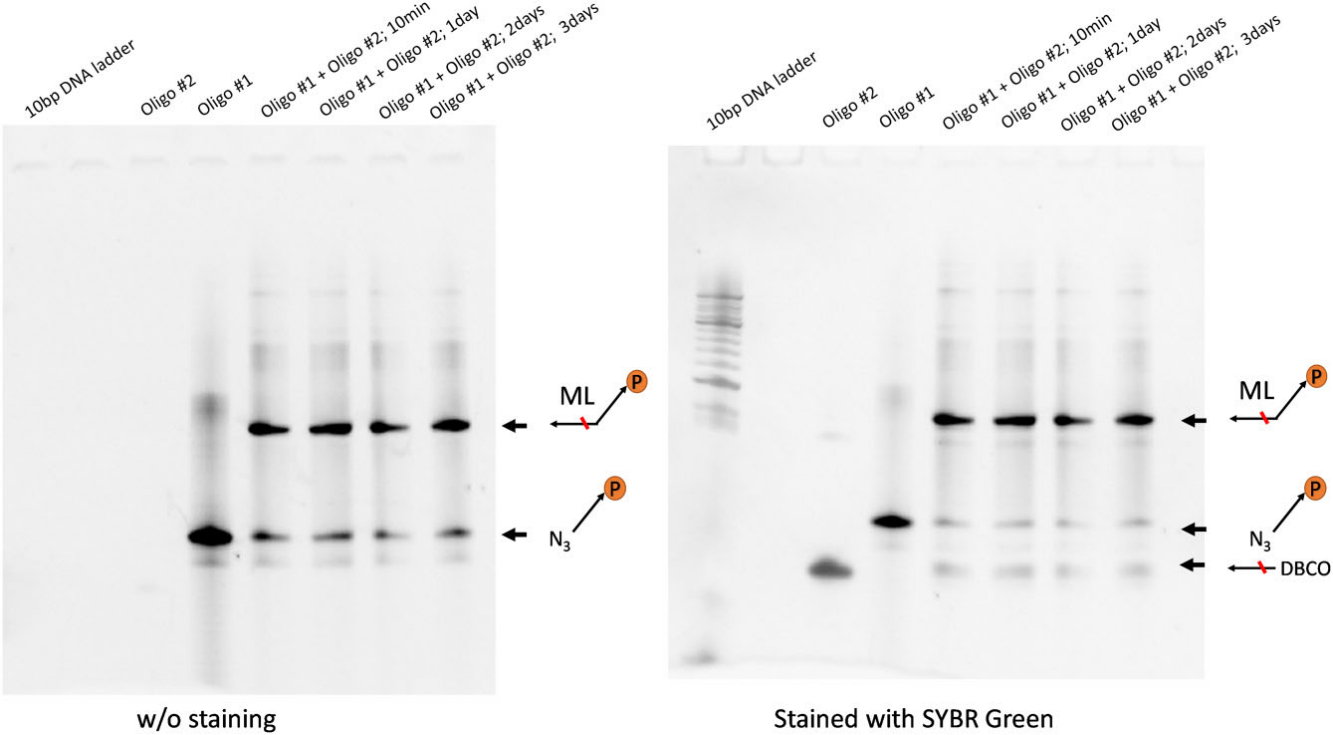


These corrections do not affect the results, discussion and conclusions presented in the article. These details have been corrected only in this correction notice to preserve the published version of record.

## Supplementary data

Supplementary Data are available at NAR Online.

## Supplementary Material

gkae1172_Supplemental_File

