# Supplementary material for: Click display: a rapid and efficient in vitro protein display method for directed evolution
Source: Nucleic Acids Res. 2023 Aug 7;51(16):e89. doi: 10.1093/nar/gkad643 (PMC10484664; doi:10.1093/nar/gkad643)
Supplement: gkad643_Supplemental_Files [file gkad643_supplemental_files.zip › Supplementary Material.pdf]

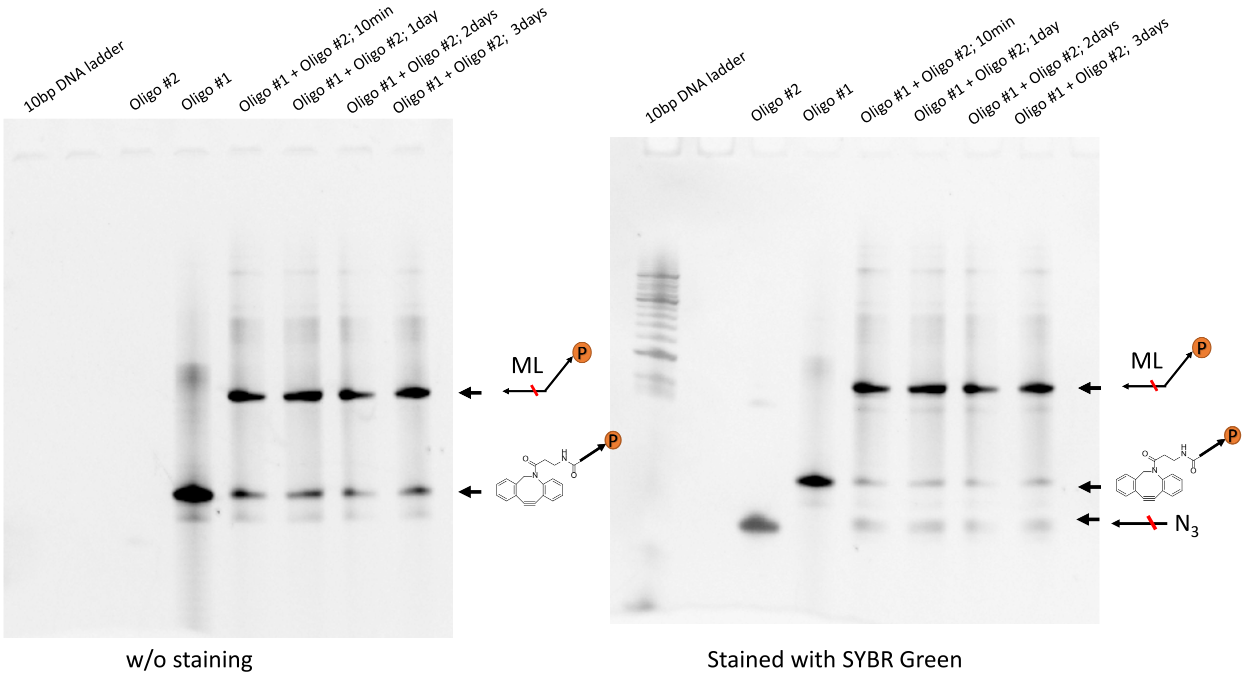

Figure S1. Gel analysis of ML synthesis reaction. Oligo #1 and Oligo #2 were mixed in at equal molar ratio (250 μM of each oligo) and reacted at room temperature for 10 minutes or 1, 2 or 3 days. The equivalent amount of each oligo (100 pmol) was analyzed on a 15% Urea-PAGE gel that was first visualized under UV, and then stained with SYBR Green and visualized again. Only Oligo #1 and ML are visible under UV without dye staining.

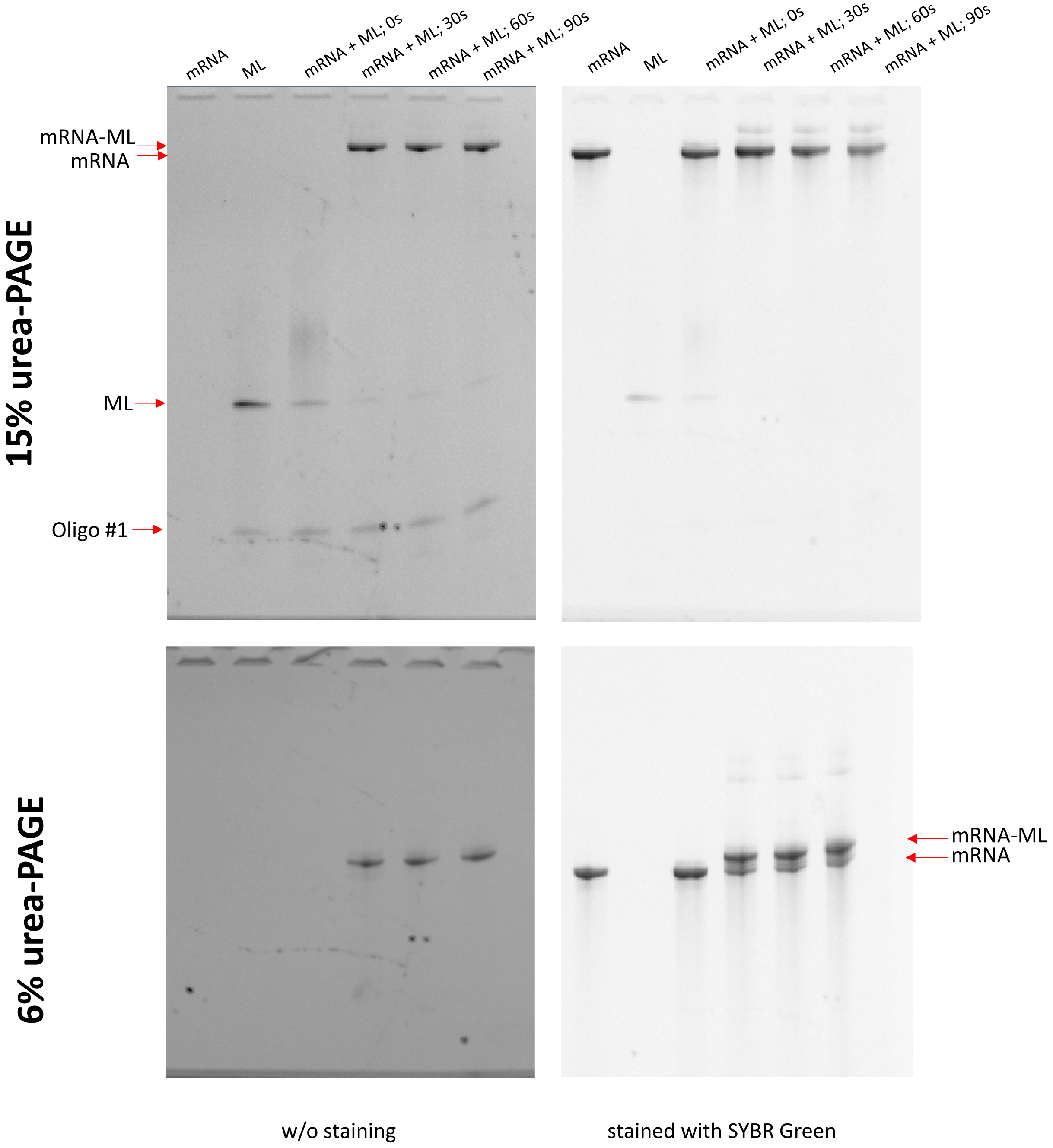

Figure S2. ML can be efficiently crosslinked to mRNA. After hybridization, the mixtures of mRNA and ML were irradiated under UV for 30, 60 or 90s before being analyzed on 15% or 6% Urea-PAGE gels. Only Oligo #1, ML and ML-crosslinked mRNA are visible on unstained gels. The band intensity for unreacted ML at 0s appears to be reduced with the appearance of a smear above the original band. This is likely due to hybridization of ML with mRNA which is partially denatured during electrophoresis.

Figure S3

DNA template encoding DARPin 3G86

Atacgaaattaatacgactcactatagg*gagacc*acaacggtttccctctagaaataattttgtttaactttaagaaggaggatatatccATGggca
gcagccaccatcaccaccatcatcatcacagcagcggcggagaacaaaaactgatcagcgaagaggatctgggatccgatctgggtaagaaactttt
ggaggcggcccgtgccgggcaagacgacgaagtgcgcattttgatggctaacggggccgacgttaacgccttggaccgctttggtttaacgcctcttc
acttggccgcacagcgcggccacttggagatcgtcgaagtcttactgaaatgtggcgctgacgtgaacgccgcagacttgtggggacaaacgccactt
catttagcagccactgctggacacttggaaattgttgaggtgttacttaaatatggggcggatgttaatgcgcttgacttaatcgggaaaacaccactg
cacctgacggcaatcgacggacatttagaaattgtggaggtgttgttaaaacatggtgcagatgtcaatgcacaggataagtttgggaagacggcgtt
cgatatctccattgataacgggaacgaggatttagcagaaatccttcaaggtaccggaggtcctactctcctcaaggaaggcaaaaggacgggggc
ggcgtggaataactagcataaccccttggggcctctaaacgggtcttgaggggttt

T7 promoter

BsaI recognition sequence

Ribosome binding site

ATG: start codon

T7 terminator

ML annealing site

Amino acid sequence of eGFP

MVSKGEELFTGVVPILVELDGDVNGHKFSVSGEGEGDATYGKLTLKFICTTGKLPVPWPTLVTTLTYGVQCFSRYPDHM
KQHDFFKSAMPEGYVQERTIFFKDDGNYKTRAEVKFEGDTLVNRIELKGIDFKEDGNILGHKLEYNYNSHNVYIMADKQ
KNGIKVNFKIRHNIEDGSVQLADHYQQNTPIGDGPVLLPDNHYLSTQSALSKDPNEKRDHMVLLEFVTAAGITLGMDEL
LEHHHHHH

Amino acid sequence of ClfA

HHHHHHGTDITNQLTNVTVGIDSGTTVYPHQAGYVKLNYGFSVPNSAVKGDTFKITVPKELNLNGVTSTAKVPPIMAG
DQVLANGVIDSDGNVIYTFTDYVNTKDDVKATLTMPAYIDPENVKKTGNVTLATGIGSTTANKTVLVDYEKYGKFYNLSI
KGTIDQIDKTNNTYRQTIYVNPSGDNVIAPVLTGNLKPNTDSNALIDQQNTSIKVYKVDNAADLSESYFVNPENFEDVTN
SVNITFPNPNQYKVEFNTPDDQITTPYIVVVNGHIDPNSKGDLALRSTLYGYNSNIIWRSMSWDNEVAFNNGSGSGDGI
DKPVVP

6xHis tag

Amino acid sequence of DARPin 1E5

MGSSHHHHHHSSGLVPRGSHMEQKLISEEDLGSDLGKKLLEAASAGQDDGVRILMANGADVNAWSHVGVTPLHLAA
AWGHLEVVEVLLKDGADVNAAIFIGHTPLHLAAARGHLEIVEVLLKNGADVNARTSAGNTPLHLAATLGHLEIVEVLLKY
GADVNAQMLYGLTAFDISIDNGNEDLAEILQ

Myc-tag

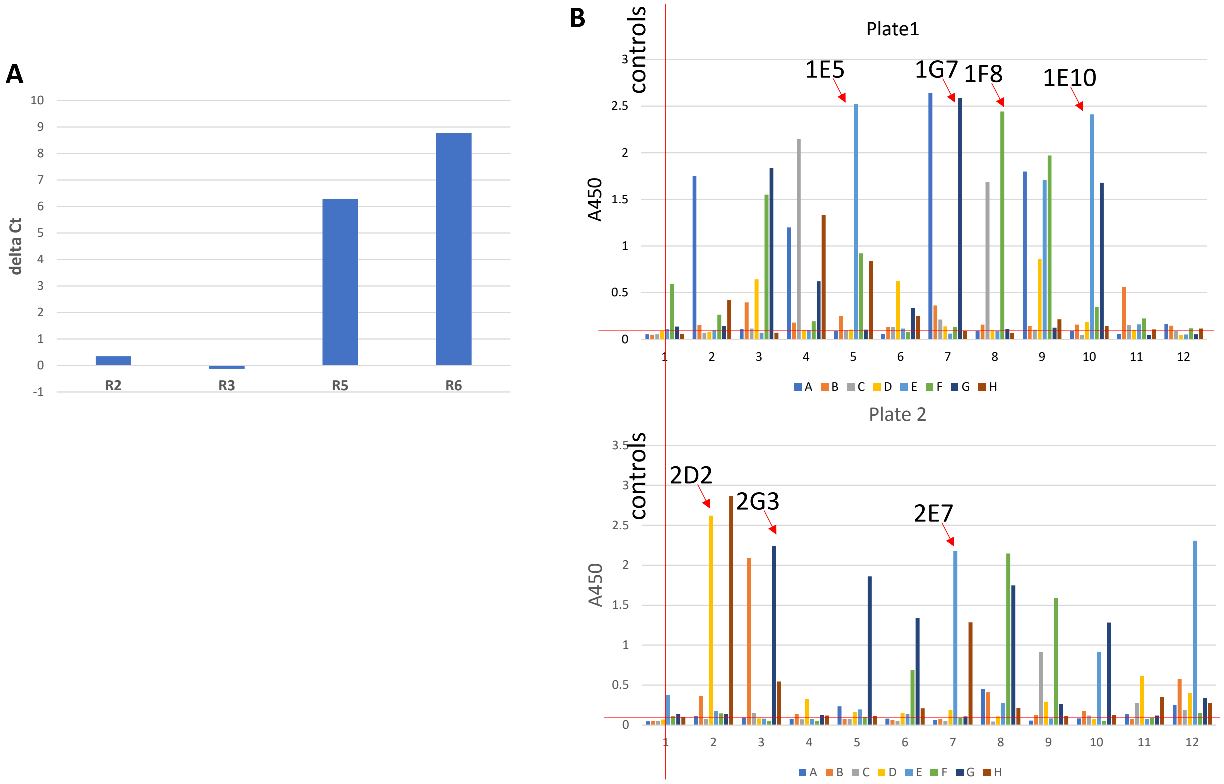

Figure S4. (A) Ct differences (ΔCt) between samples from representative rounds incubated in the absence and presence ClfA. **(B)** ELISA-based screen to identify individual DARPin molecules able to bind ClfA.

Motif	NCAP / AR1 / AR2 / AR3 / CCAP
	GKKLLEAARAGQDDEVRILMANGADVNA/XXXXGXTPLHLAAXXGHLEIVEVLLKXGADVNA/XXXXGXTPLHLAAXXGHLEIVEVLLKXGADVNA/XXXXGXTPLHLAAXXGHLEIVEVLLKXGADVNA/QXXXGXTAFDISIDNGNEDLAEILQ
P1E5	GKKLLEAASAGQDDGVRILMANGADVNA/WSHVGVTPLHLAAAWGHLEVVEVLLKDGADVNA/AIFIGHTPLHLAAARGHLEIVEVLLKNGADVNA/RTSAGNTPLHLAATLGHLEIVEVLLKYGADVNA/QMLYGLTAFDISIDNGNEDLAEILQ
P1E10	GKKLLEAARAGQDDEARILMANGADVNA/YTVLGITPLHLAAFNGHLEIVEVLLNDGADVNA/LSAGGVTPLHLAALCGHLEIVEVLLKHGADVNA/REWFGFTPLHLAATRGHLEIVEVLLKNGADVNA/QPPDGNTAFDIPIDNGNEDLAEILQ
P1F8	GKKLLEAARAGQDDGARILMANGADVNA/YTVLGITPLHLAAFNGHLEIVEVLLNDGADVNA/LSAGGVTPLHLAALCGHLEIVEVLLKHGADVNA/REWFGFTPLHLAATRGHLEIVEVLLKHGADVNA/QTPLGHTAFDISIDNGNEDLAEILQ
P1G7	GKKLLEAARAGQDDEVHILMANGADVNA/YTVLGITPLHLAAFNGHLEIVEVLLKDGADVNA/LSAGGVTPLHLAALCGHLEIVEVLLKHGADVNA/REWFGFTPLHLAATRGHLEIVEVLLKHGADVNA/QPAVGTTAFDISIGNGNEDLAEILQ
P2D2	GKKLLEAARAGQDDEVRILMANGADVNA/YTVLGITPLHLAAFNGHLETVEVLLKDGADVNA/LSAGGVTPLHLAALCGHLEIVEVLLKHGADVNA/REWFGFTPLHLAATRGHLEIVEVLLKNGADVNA/QPPDGNTAFDIPTDNGNEDLAEILQ
P2G3	GKKLLEAARAGQDDEVRILMANGADVNA/YTVLGITPLHLAAFNGHLEIVEVLLKDGADVNA/LSAGGVTPLHLAALCGHLEIVEVLLKHGADVNA/REWFGFTPLHLAATRGHLEIVEVLLKNGADVNA/QPPDGNTAFDISIDNGNEDLAEILQ
P2E7	GKKLLEAARAGQDDEVRILMANGADVNA/YTVLGITPLHLAAFNGHLEIVEVLLKDGADVNA/LSAGGVTPLHLAALCGHLEIVEVLLKHGADVNA/REWFGFTPLHLAATRGHLEIVEVLLKNGADVNA/QPPDGNTTFDISIDNGNEDLAEILQ

Figure S5. Sequences of the selected ClfA-binding DARPins from ELISA screen.

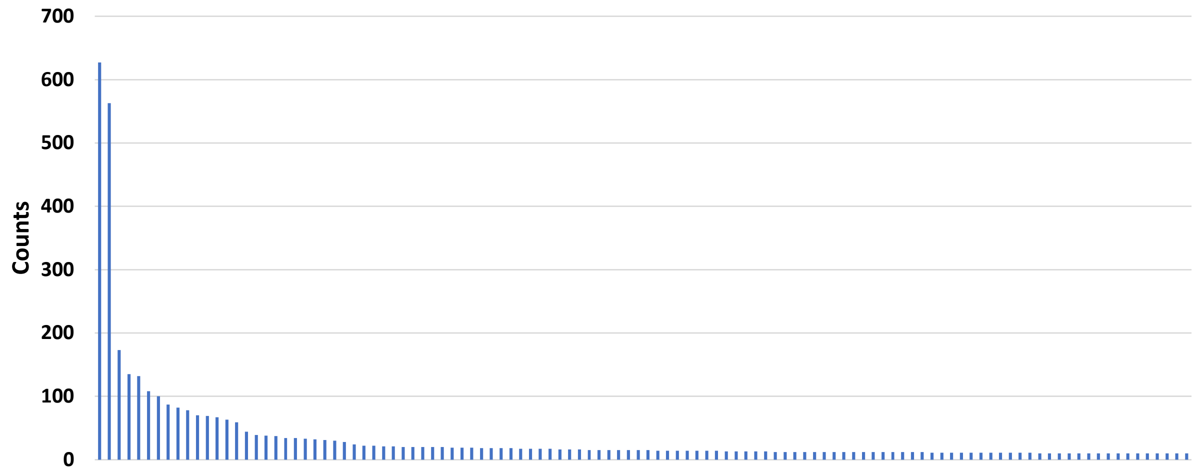

Figure S6. Counts of different DARPin sequences from the R6 library.

Table S1
Primer sequences:

/i3CyAn/: CynK
/i5-TAMK/: Int 5-TAMRA

Name	Sequence	Vendor
Oligo #1	/5DBCON/AAATTCCA/i3Cyan/GCCGCCCCCCGTCCT	IDT
Oligo #2	/5AzideN/AAAA/i5-TAMK/AAAAAAAAAAAAAAAA AAAACC/3Puro/	IDT
1892	5- (PHOSPHORYLATED)TTG AGG AGA GTA GGA CCT	Sigma
2488	GTT TAA CTT TAA GAA GGA GGA TAT ATC CAT	Sigma
1938	ATA CGA AAT TAA TAC GAC TCA CTA TAG GGA GAC CAC AAC GGT TTC CCT CTA GAA ATA ATT TTG TTT AAC	Sigma
2643	AAA CCC CTC CGT TTA GAG AGG GGT TAT GCT AGT TAT TCC ACG CCG CCC CCC GTC CT	Sigma
AR-F2	AAAAAGGTCTCACGCCNNKNNKNNKNNKGGTNNKACGCCT CTTCACTTGGCCGCANNKNNKGGCCACTTGGAGATCGTCGA AGTCTTACTGAAANNKGGCGCTGACGTGAACGCCNNKNNK NNKNNKGGANNKACGCCACTTCATTTAGCAGCCNNKNNKG GACACTTGGAAATTGTTGAGGTGTTACTTAAA	IDT
AR-R2	TTTTTGGTCTCTCCGTMNNCCCMNNMNNMNNCTGTGCAT TGACATCTGCACCMNNTTTTAACAACACCTCCACAATTTCTA AATGTCCMNNMNNTGCCGCCAGGTGCAGTGGTGTMNNC CCMNNMNNMNNMNNCGCATTAACATCCGCCCCMNNTTT AAGTAACACCTCAACAATTTCCAAGTGTCC	IDT
2617	AAA AAC TCG AGA TAC GAA ATT AAT ACG ACT CAC TAT AGG GTG ACC ACA ACG GTT TCC CTC	Sigma
2701	AAA AAG GTC TCA GGC GTT AAC GTC GGC CCC GTT AGC	Sigma
2702	AAA AAG GTC TCA ACG GCG TTC GAT ATC TCC ATT GA	Sigma
2581	AAA AAC TCG AGT TAT TCC ACG CCG CCC C	Sigma
DARPin_NGS_F0	ACACTCTTTCCCTACACGACGCTCTTCCGATCT aa atctgggatccgacctgggt	Sigma
DARPin_NGS_F1	ACACTCTTTCCCTACACGACGCTCTTCCGATCT ac atctgggatccgacctgggt	Sigma
DARPin_NGS_F2	ACACTCTTTCCCTACACGACGCTCTTCCGATCT at atctgggatccgacctgggt	Sigma
DARPin_NGS_F3	ACACTCTTTCCCTACACGACGCTCTTCCGATCT ag atctgggatccgacctgggt	Sigma
DARPin_NGS_R	GACTGGAGTTCAGACGTGTGCTCTTCCGATCT GTA CCC GAG CTC GAT TGC AG	Sigma

/i3Cyan/: internal CNVK oligo is from Glen Research (https://www.glenresearch.com/10-4960.html)
/3Puro/: 3' Puromycin
/5DBCON/: 5' Dibenzocyclooctyl (DBCO)
/5AzideN/: 5' Azide (NHS Ester)
