# Supplementary figures and images for: Click display: a rapid and efficient in vitro protein display method for directed evolution
Source: Nucleic Acids Res. 2023 Aug 7;51(16):e89. doi: 10.1093/nar/gkad643 (PMC10484664; doi:10.1093/nar/gkad643)

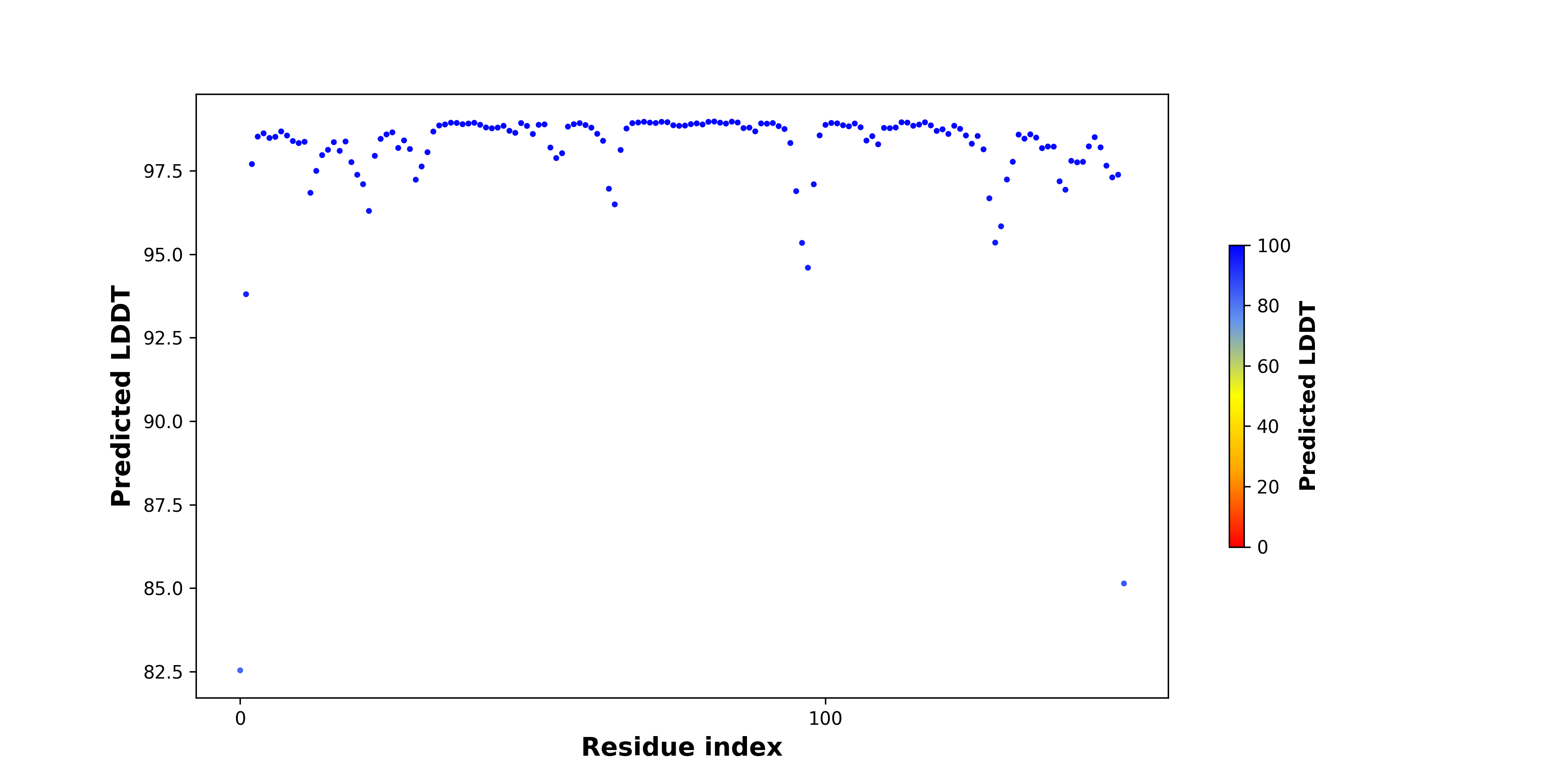

Supplement: gkad643_Supplemental_Files [file gkad643_supplemental_files.zip › ranked_0_pLDDT.png]
