# Supplementary material for: Correction to ‘Click display: a rapid and efficient in vitro protein display method for directed evolution’
Source: Nucleic Acids Res. 2024 Nov 18;52(22):14280–1. doi: 10.1093/nar/gkae1172 (PMC11662653; doi:10.1093/nar/gkae1172)
Supplement: gkae1172_Supplemental_File [file gkae1172_supplemental_file.pdf]

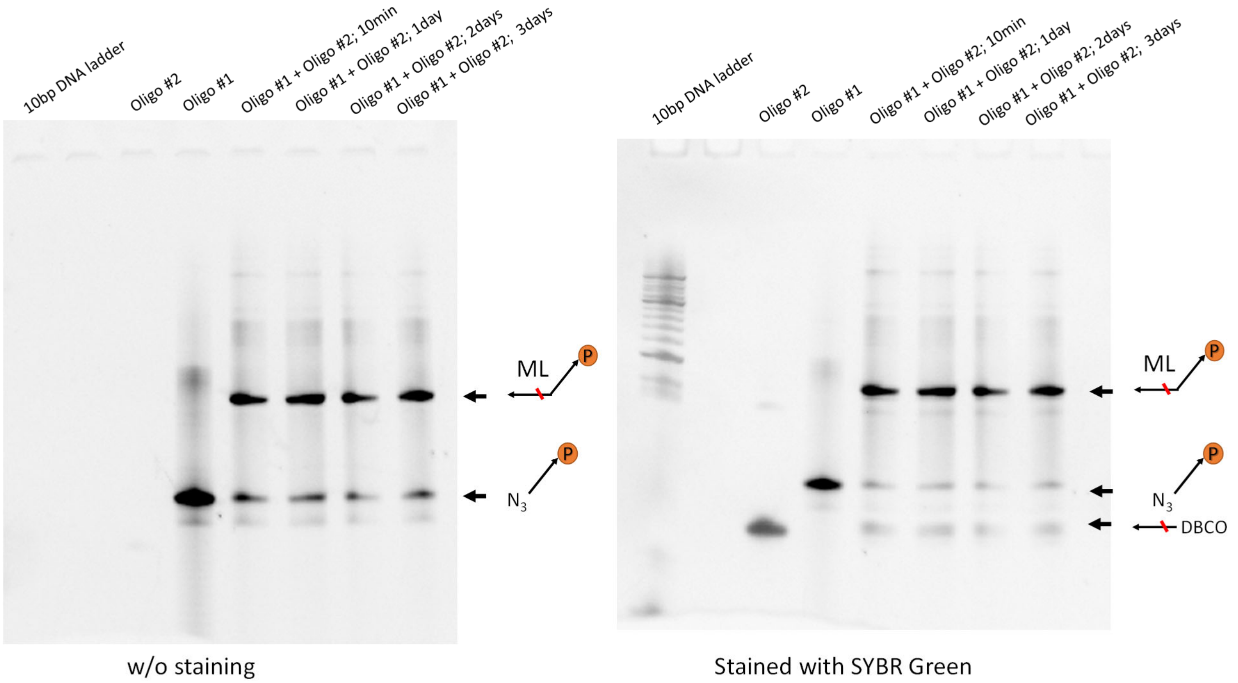

Figure S1. Gel analysis of ML synthesis reaction. Oligo #1 and Oligo #2 were mixed in at equal molar ratio (250 µM of each oligo) and reacted at room temperature for 10 minutes or 1, 2 or 3 days. The equivalent amount of each oligo (100 pmol) was analyzed on a 15% Urea-PAGE gel that was first visualized under UV, and then stained with SYBR Green and visualized again. Only Oligo #1 and ML are visible under UV without dye staining.

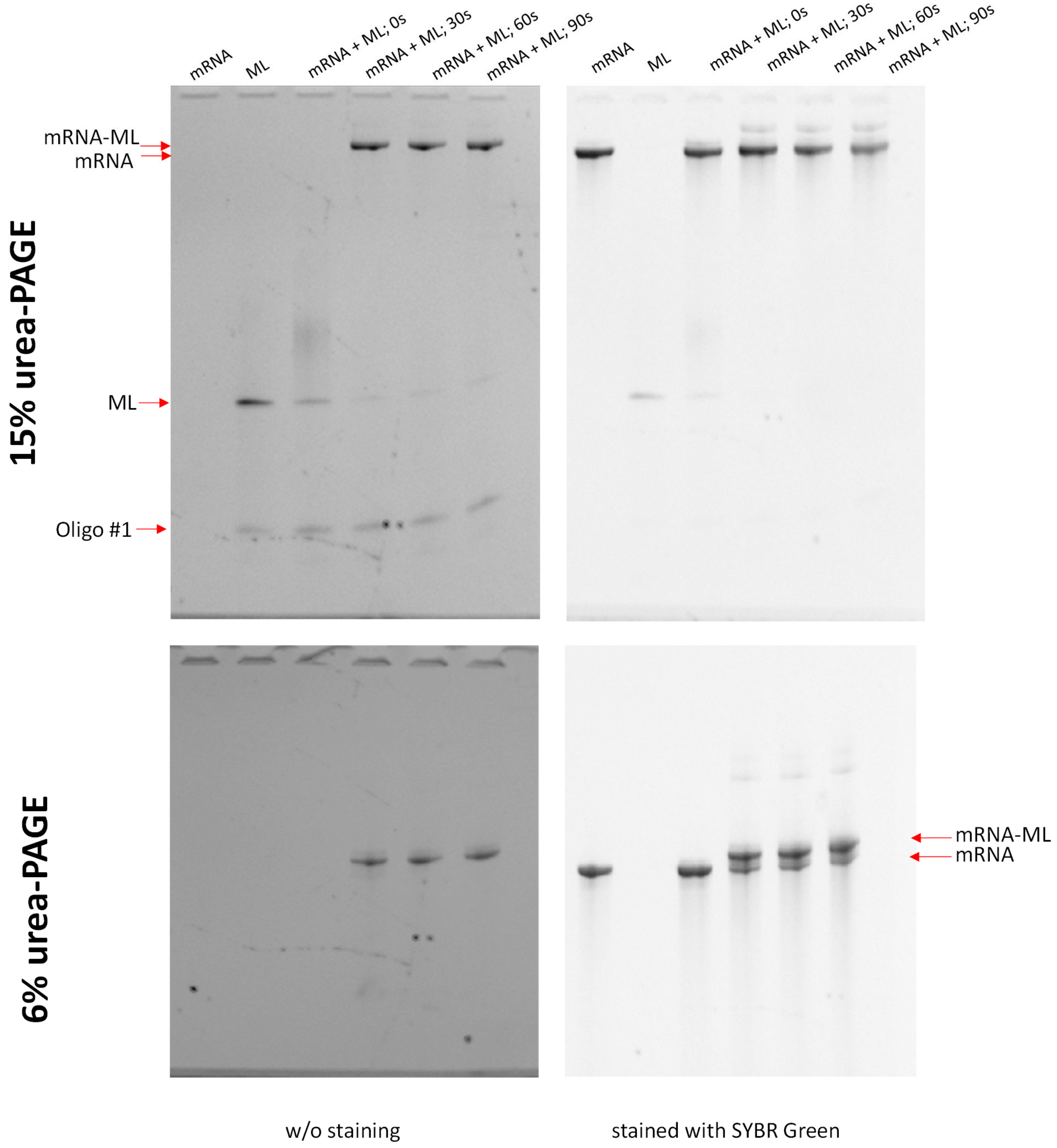

Figure S2. ML can be efficiently crosslinked to mRNA. After hybridization, the mixtures of mRNA and ML were irradiated under UV for 30, 60 or 90s before being analyzed on 15% or 6% Urea-PAGE gels. Only Oligo #1, ML and ML-crosslinked mRNA are visible on unstained gels. The band intensity for unreacted ML at 0s appears to be reduced with the appearance of a smear above the original band. This is likely due to hybridization of ML with mRNA which is partially denatured during electrophoresis.

Figure S3

DNA template encoding DARPin 3G86

Atacgaaattaatacgactcactatagg*gagacc*acaacggtttccctctagaaataattttgtttaactttaagaaggaggatatatccATGggca
gcagccaccatcaccaccatcatcatcacagcagcggcggagaacaaaaactgatcagcgaagaggatctgggatccgatctgggtaagaaactttt
ggaggcggcccgtgccgggcaagacgacgaagtgcgcattttgatggctaacggggccgacgttaacgccttggaccgctttggtttaacgcctcttc
acttggccgcacagcgcggccacttggagatcgtcgaagtcttactgaaatgtggcgctgacgtgaacgccgcagacttgtggggacaaacgccactt
catttagcagccactgctggacacttggaaattgttgaggtgttacttaaatatggggcggatgttaatgcgcttgacttaatcgggaaaacaccactg
cacctgacggcaatcgacggacatttagaaattgtggaggtgttgttaaaacatggtgcagatgtcaatgcacaggataagtttgggaagacggcgtt
cgatatctccattgataacgggaacgaggatttagcagaaatccttcaaggtaccggaggtcctactctcctcaaggaaggcaaaaggacgggggc
ggcgtggaataactagcataaccccttggggcctctaaacgggtcttgaggggttt

T7 promoter

Bsal recognition sequence

Ribosome binding site

ATG: start codon

T7 terminator

ML annealing site

Amino acid sequence of eGFP

MVSKGEELFTGVVPILVELDGDVNGHKFSVSGEGEGDATYGKLTLKFICTTGKLPVPWPTLVTTLTYGVQCFSRYPDHM
KQHDFFKSAMPEGYVQERTIFFKDDGNYKTRAEVKFEGDTLVNRIELKGIDFKEDGNILGHKLEYNYNSHNVYIMADKQ
KNGIKVNFKIRHNIEDGSVQLADHYQQNTPIGDGPVLLPDNHYLSTQSALSKDPNEKRDHMVLLEFVTAAGITLGMDEL
LEHHHHHH

Amino acid sequence of ClfA

HHHHHHGTDITNQLTNVTVGIDSGTTVYPHQAGYVKLNYGFSVPNSAVKGDTFKITVPKELNLNGVTSTAKVPPIMAG
DQVLANGVIDSDGNVIYTFTDYVNTKDDVKATLTMPAYIDPENVKKTGNVTLATGIGSTTANKTVLVDYEKYGKFYNLSI
KGTIDQIDKTNNTYRQTIYVNPSGDNVIAPVLTGNLKPNTDSNALIDQQNTSIKVYKVDNAADLSESYFVNPENFEDVTN
SVNITFPNPNQYKVEFNTPDDQITTPYIVVVNGHIDPNSKGDLALRSTLYGYNSNIIWRSMSWDNEVAFNNGSGSGDGI
DKPVVP

6xHis tag

Amino acid sequence of DARPin 1E5

MGSSHHHHHHSSGLVPRGSHMEQKLISEEDLGSDLGKKLLEAASAGQDDGVRILMANGADVNAWSHVGVTPLHLAA
AWGHLEVVEVLLKDGADVNAAIFIGHTPLHLAAARGHLEIVEVLLKNGADVNARTSAGNTPLHLAATLGHLEIVEVLLKY
GADVNAQMLYGLTAFDISIDNGNEDLAEILQ

Myc-tag

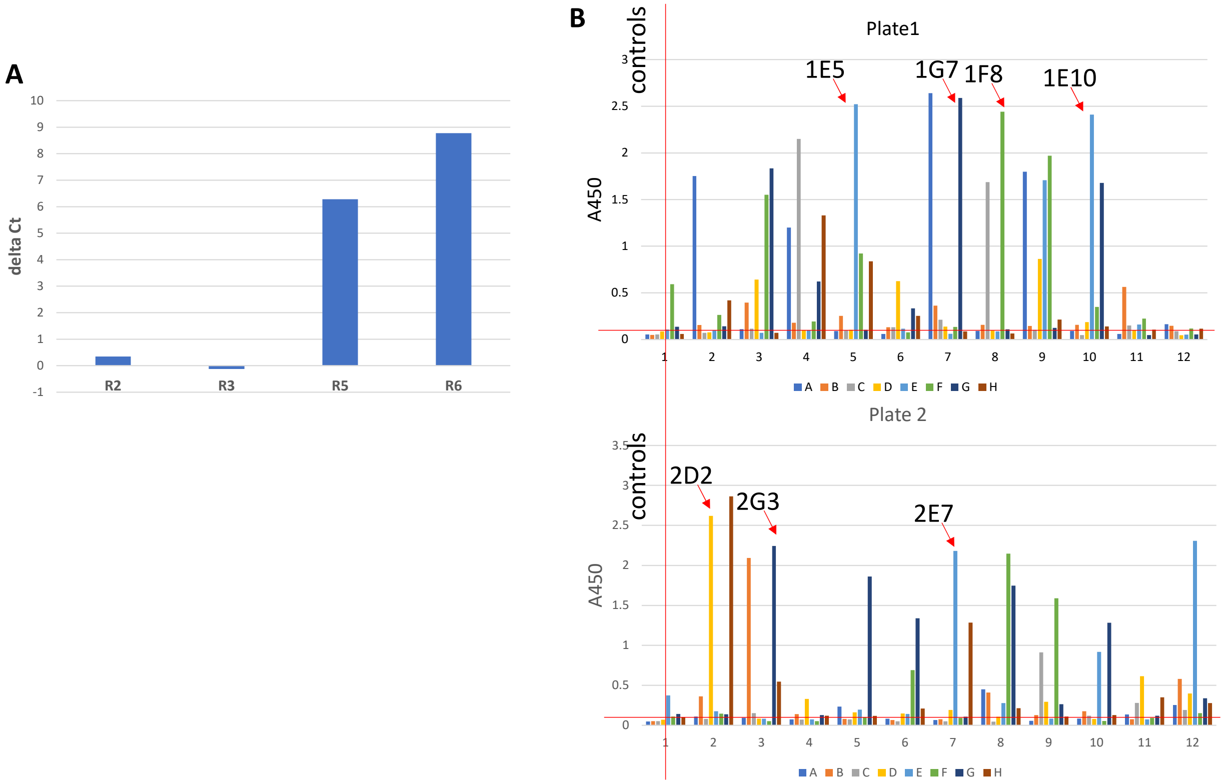

Figure S4. (A) Ct differences (ΔCt) between samples from representative rounds incubated in the absence and presence ClfA. **(B)** ELISA-based screen to identify individual DARPin molecules able to bind ClfA.
